# Data on changes in flexural strength and elastic modulus of dental CAD/CAM composites after deterioration tests

**DOI:** 10.1016/j.dib.2019.103889

**Published:** 2019-04-03

**Authors:** Hiroshi Ikeda, Yuki Nagamatsu, Hiroshi Shimizu

**Affiliations:** Division of Biomaterials, Department of Oral Functions, Kyushu Dental University, Fukuoka 803-8580, Japan

**Keywords:** Dental restorative material, Composite, Flexural strength, Elastic modulus, Deterioration tests

## Abstract

Mechanical properties of dental restorative materials are important for clinical success in prosthodontic care. However, open data on the mechanical properties of these materials are limited. This article provides data on the flexural strength and elastic modulus of dental composites in practical use for design/computer-aided manufacturing (CAD/CAM) systems. Eight brands of composites were subjected to deterioration tests: immersing in water at 37 °C for one day or seven days, or thermocycling (TC) in water at temperatures between 5 °C and 55 °C for 5000 or 10,000 cycles. The mechanical properties of the samples were measured by using a three-point bending test according to ISO 6872. The obtained values were statistically analyzed using one-way analysis of variance (ANOVA), followed by Tukey multiple comparison tests.

**Table:** Specifications table

Subject area	Dentistry, Material Science
More specific subject area	Dental Materials
Type of data	2 Figures, 1 Table
How data was acquired	Three-point bending test (ISO 6872)
Data format	Raw and statistically analyzed data
Experimental factors	Two types of the deterioration tests are employed. One involves immersing the samples in water at 37 °C for one day or seven days. The other involves subjecting the samples to 5000 or 10,000 cycles of thermocycling in water baths at temperatures between 5 °C and 55 °C.
Experimental features	Flexural strength and elastic modulus of eight brands of commercially dental CAD/CAM composites were measured after the deterioration tests.
Data source location	Division of Biomaterials, Department of Oral Functions, Kyushu Dental University 2-6-1 Manazuru, Kokura-kitaku, Kitakyushu, Fukuoka 803–8580, Japan
Data accessibility	All data herein and supplementary files are available within this article.
Related research article	None.

**Table:** 

**Value of the data** • The data can help obtain the mechanical properties of the dental composites in practical use. • The data can be helpful for dentists to choose the dental CAD/CAM composites for clinical treatment. • The data can be useful for developing dental materials. • The data can be compared with that on dental restorative materials, including ceramics and composites.

## 1 Data

**Fig. 1:**
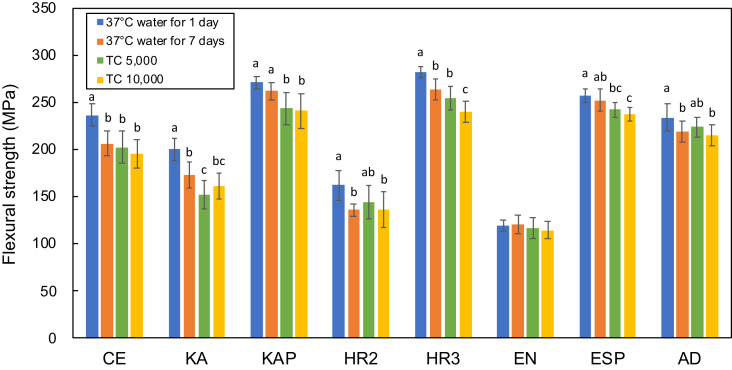
Flexural strength of the dental CAD/CAM composites after each deterioration test. Different letters represent a statistically significant difference between the groups for each composite (p < 0.05).

**Fig. 2:**
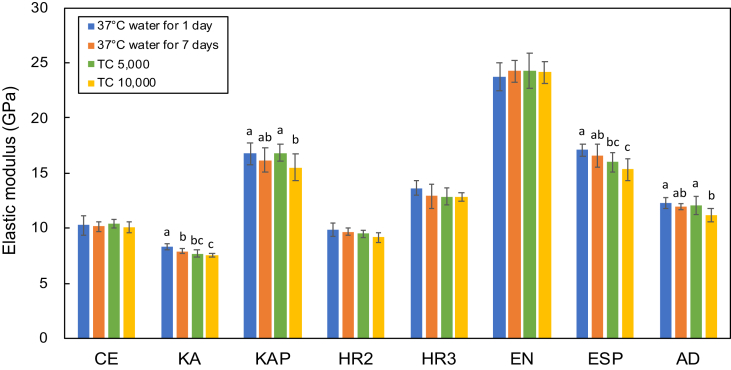
Elastic moduli of the dental CAD/CAM composites after each deterioration test. Different letters represent a statistically significant difference between the groups for each composite (p < 0.05).

The datasets provide information on the mechanical properties of dental CAD/CAM composites in clinical use. Table 1 lists eight brands of commercial composites used in this experimental study. Fig. 1, Fig. 2 show the flexural strength and elastic moduli, respectively, of the composites after each deterioration test. Changes in the values due to the deterioration tests are statistically analyzed in each composite by ANOVA, followed by Tukey multiple comparison tests; the results are shown in Fig. 1, Fig. 2.

**Table 1:** The CAD/CAM composites used in this experiment.

Product name	Abbreviation	Manufacturer	LOT	Ref.
Cerasmart 300	CE	GC Corp.	1711072	[1]
KATANA AVENCIA Block	KA	Kuraray Noritake Dental Inc.	000126	[2]
KATANA AVENCIA P Block	KAP	Kuraray Noritake Dental Inc.	000019	[3]
KZR-CAD HR 2	HR2	YAMAKIN Co., Ltd.	01061807	[4]
KZR-CAD HR 3	HR3	YAMAKIN Co., Ltd.	01061822	[5]
VITA ENAMIC	EN	VITA Zahnfabrik H. Rauter GmbH & Co. KG	77430	[6]
ESTELITE P BLOCK	ESP	Tokuyama Dental Corp.	0030Y8	[7]
ARTESANO DUR	AD	Yamahachi Dental MFG., Co.	NF13R	[8]

## 2 Experimental design, materials and methods

### 2.1 Sample preparation

The as-received composite blocks were cut into bar shapes of dimensions 14 mm × 4 mm × 1.2 mm using a diamond wheel saw (model 650, South Bay Technology, USA). The bar samples were polished under dry condition by using an emery paper up to #2000. The edges of the samples were chamfered by polishing. The prepared samples were subjected to the deterioration test, either the water immersion test or the thermocycling one. The former was performed by immersing the samples in water at 37 °C for one day or seven days. The latter was conducted by immersing the samples in water baths at temperatures between 5 °C and 55 °C for 5000 or 10,000 cycles with a 20s dwell time at each temperature. The thermocycle test has been widely used to simulate the physiological aging experienced by biomaterials in clinical practice [9]. After each deterioration test, the samples were subjected to the three-point bending test.

### 2.2 Three-point bending test

According to ISO 6872, the flexural strength and elastic modulus of the composite samples after each deterioration test were measured by a three-point bending test, performed at room temperature using a universal testing machine (AGS-H, Shimadzu Corp., Japan) at a crosshead speed of 1 mm/min and with 12-mm supporting span. The flexural strength (σ) was calculated using the following equation,

$$\sigma =\;3\text{FL}/{2\text{bh}}^{2}$$

where F is the maximum load during the bending test, L is the supporting span, b is the width of the sample, h is the thickness of the sample. The elastic modulus (E) was calculated from the three-point bending test result using the following equation,

$$\text{E}\;=\;{\text{mL}}^{3}/{4\text{bh}}^{3}$$

where, m is the gradient of the initial straight-line portion of the load deflection.

### 2.3 Statistical analysis

The results of the flexural strength and elastic modulus were analyzed using a statistical software EZR (Saitama Medical Center, Japan). The mean and standard deviation were calculated by using n = 10 raw data. The one-way analysis of variance (ANOVA), followed by Tukey's post-hoc test, was performed for the multiple comparisons between the groups. The significance level was set at 0.05 for all analyses.
